# Guidelines for the Management of Complications of Diabetes in Saudi Arabia Using Delphi Technique for Consensus Among National Experts

**DOI:** 10.3390/clinpract15010009

**Published:** 2024-12-31

**Authors:** Raed Aldahash, Mohammed A. Batais, Ashraf El-Metwally, Saja Alhosan, Mohammed Alharbi, Mohammed Almutairi, Abdulghani Alsaeed, Mohammed Alsofiani, Mohammed AlMehthel, Mohammed Aldubayee, Khaled Aldossari, Sulieman Alshehri

**Affiliations:** 1 Department of Medicine, Ministry of National Guard Health Affairs, Riyadh 11426, Saudi Arabia; dr.raaed@hotmail.com; 2 King Abdullah International Medical Research Center, King Saud bin Abdulaziz University for Health Sciences, Riyadh 11481, Saudi Arabia; m.aldubayee@gmail.com; 3 Department of Family and Community Medicine, College of Medicine, King Saud University, Riyadh 12372, Saudi Arabia; drmohammed34@gmail.com; 4 University Diabetes Center, King Saud University Medical City, Riyadh 12372, Saudi Arabia; 5 Saudi National Diabetes Center, Saudi Health Council, Riyadh 13315, Saudi Arabia; szalhosan@gmail.com (S.A.); s.alshehri@shc.gov.sa (S.A.); 6 Deputyship for Therapeutic Services, Ministry of Health, Riyadh 12613, Saudi Arabia; myh.alharbi24@gmail.com; 7 Internal Medicine Department, Security Forces Hospital, Riyadh 11481, Saudi Arabia; dr_mutairi78@hotmail.com; 8 Prince Sultan Military Medical City, Riyadh 11159, Saudi Arabia; draalsaeed@hotmail.com; 9 Endocrinology Unit, Internal Medicine Department, College of Medicine, King Saud University, Riyadh 12372, Saudi Arabia; malsofiani@ksu.edu.sa; 10 Division of Endocrinology, Diabetes and Metabolism, Johns Hopkins University, Baltimore, MD 21287, USA; 11 Obesity, Diabetes and Endocrinology Center, King Fahad Medical City, Riyadh 12231, Saudi Arabia; dr_almehthel@hotmail.com; 12 Division of Endocrinology, University of British Columbia, Vancouver, BC V5Z 1M9, Canada; 13 Department of Pediatrics, Ministry of National Guard Health Affairs, Riyadh 11426, Saudi Arabia; 14 Department of Family and Community Medicine, College of Medicine, Prince Sattam Bin Abdulaziz University, Al-Kharj 11942, Saudi Arabia; khalid317@gmail.com

**Keywords:** diabetes, diabetes complications, diabetes screening, diabetes management, guidelines, Delphi technique

## Abstract

(1) Background: Saudi Arabia has one of the leading cases of diabetes globally, with approximately 27.8% of adults suffering from the disease. Given the negative consequences of diabetes mellitus (DM), it is critical to develop guidelines for its management. (2) Methods: After a thorough review of the literature around diabetes management, a diverse panel of 14 clinical experts was identified to participate in the Delphi process. The Delphi process included three rounds to ensure all available evidence was accounted for. (3) Results: The Delphi method concluded with a total of 37 guidelines reviewed and approved by the panelists, followed by verification from a third party in Saudi Arabia. The Delphi and external evaluation confirmed that authentic, relevant, and applicable evidence for diabetes management in Saudi Arabia was accounted for. The process concluded with a list of 37 statements about the management of acute and chronic complications of diabetes in Saudi Arabia. (4) Conclusions: The preparation of contextual evidence for the management of diabetes in Saudi Arabia will be instrumental in addressing the burden of disease in the region. The guidelines offer useful insights into diabetes care, especially by prioritizing early detection and proactive management of complications. They highlight the importance of lifestyle changes and medical therapy. However, due to the ever-changing nature of diabetes, the document must be monitored and updated on a regular basis to ensure its continued relevance and effectiveness.

## 1. Introduction

Diabetes mellitus, a prevalent and chronic metabolic disorder characterized by hyperglycemia, affects a significant portion of the global population [1]. Diabetes prevalence is expected to skyrocket by 2040, with an estimated 642 million people suffering from the disease, up from 415 million in 2015 and 151 million in 2000 [2]. A comprehensive assessment of the impact of diabetes mellitus reveals that it has a negative impact on the many aspects of an individual’s quality of life, including physical health, economic stability, and social acceptance [3]. The east and north African regions have an alarmingly high prevalence of diabetes, estimated at around 12.2%. This major public health concern is clearly associated with increased morbidity and mortality rates [4]. Among Middle Eastern nations, Saudi Arabia ranks second in terms of diabetes prevalence, while on a global scale, it occupies the seventh position. Notably, a community-based national epidemiological health survey revealed a diabetes prevalence of 23.7% [5]. Matching global trends, Saudi Arabia’s escalating diabetes prevalence raises vulnerability to its harmful effects [6].

Type 2 diabetes accounts for more than 90% of the 425 million adults with diabetes [7]. Diabetes mellitus has a profound and multifaceted impact on adults’ health and well-being, affecting the physical, social, and economic spheres [8]. Thus, Diabetes mellitus control programs ought to be considered as a higher priority for the health ministry. Moreover, it is a major public health concern in Saudi Arabia and has a significant impact on the health of the population [3]. Sedentary lifestyles, high-fat diets, and widespread overweight/obesity all contribute significantly to Saudi Arabia’s high diabetes burden [9].

Diabetic metabolic disturbances have a wide range of consequences, including macro/microvascular dysfunction and increased risks of cardiovascular disease, stroke, and amputation. Critical collaboration is required to mitigate these risks and protect young adults from the effects of diabetes [10]. Global diabetes is a significant public health threat, as evidenced by a previous study finding that nearly 10% of diabetics worldwide are at an increased risk of having their first myocardial infarction [11]. Compared to non-diabetics, diabetic patients face a significantly elevated risk of lower extremity amputation, 17 to 40 times higher [12]. Furthermore, diabetic individuals are prone to non-healing ulcers and wounds, while high blood sugar and smoking further exacerbate foot problems and amputation risks [13]. Diabetes risk reduction necessitates proactive measures such as proper diabetes management, dietary changes, avoiding excessive exercise, and quitting smoking, alongside consistent blood sugar management [14,15].

A Delphi method is used to address the challenge of diabetes control, leveraging the combined expertise of stakeholders, experts, and professionals through iterative rounds of questionnaires and surveys [16]. This method sought a collaborative consensus on key aspects of addressing type 2 diabetes, such as identifying research gaps, developing training and curriculum for priority health worker competencies, and establishing measurable behavioural change goals. It also aims to use technology, such as mobile health, to engage stakeholders and implement data-driven strategies [17]. Therefore, the effectiveness of the modified Delphi method for gathering and synthesizing professional experience and viewpoints to gather knowledge for different aspects of diabetes treatment and its control program was needed, which would impact the structure management and design tools for type 2 diabetes mellitus [18]. The guidelines were developed by a multidisciplinary team of experts in diabetes care, including endocrinologists, diabetologists, diabetes educators, and other relevant specialist [19]. Recognizing the unique challenges faced by the Saudi population, a Delphi method was used to engage national-level experts in the development of evidence-based guidelines for managing diabetes complications. This collaborative approach focuses on overcoming existing barriers and improving healthcare outcomes through iterative expert input and consensus-driven strategies. Furthermore, this research aims to provide new goals and targets to develop a set of national guidelines that would provide a comprehensive framework for diabetes treatment and its management. The study findings would reveal health reforms to overall reduce the burden of non-communicable diseases and outcomes, which can have detrimental impacts on the quality of life in Saudi Arabia.

## 2. Materials and Methods

### 2.1. Panel Selection

The Delphi method was chosen to develop national diabetes management guidelines for Saudi Arabia because of its proven effectiveness in achieving consensus among medical experts. This time-tested methodology ensures a rigorous and evidence-based approach to streamlining and organizing available knowledge [20,21]. The study recruited 14 diabetes experts from Saudi Arabia after obtaining their informed consent. Invitations were sent to a larger pool of relevant specialists, and the research included the first 14 respondents.

### 2.2. A Systematic Review of the Literature

Systematic reviews were conducted to ensure inclusion of relevant publications during the process of reviewing the guidelines. Different search terms that were used included “Diabetes”, “Diagnostic”, “Therapy”, and “Management”. This search was conducted across different search engines, including PubMed, Embase, and Cochrane Library. Additionally, grey literature was included in the search to ensure that no important evidence was missed, and a robust review was generated. Articles included were relevant to the management of diabetes, and comprised of the screening, diagnostic, and therapeutic approaches. Importance was given to randomized controlled trials, systematic review, and meta-analysis; 53 relevant studies were identified, out of which 37 recommendations were made for diabetes complications.

### 2.3. Data Extraction and Statement Development

The articles collected were entered into an Excel sheet, and the type of evidence was assessed based on the quality as well as the study design of the article. This data relevant to the articles was entered into an Excel sheet, and then each article was discussed in the context of the contribution of the article to formulate guidelines related to management of complications caused by diabetes.

### 2.4. Round 1

The first round of the Delphi method featured the distribution of a comprehensive document consisting of 37 statements, study objectives, and participation guidelines to 14 appointed panellists. Each of the 14 panel members was asked to indicate whether they agreed or disagreed with the assertions and, where appropriate, to offer comments or ideas.

### 2.5. Round 2

Statements that did not receive consensus in round 1 were resubmitted to the 14 participants via email. This second round, which used the same voting procedure as the first, added the element of anonymous group ratings and comments. This iterative process enabled panellists to revise their feedback while reflecting on the group’s collective insights.

### 2.6. Round 3

Face-to-face meetings were used in Round 3, and the acceptance or rejection of a statement was still based on 80% agreement. The show of hands was used by the panellists to facilitate discussion and reach an agreement, removing the anonymity of the responses. Once the agreement for all remaining statements was achieved, members then discussed the ideal clinical course for managing diabetes-related complications in Saudi Arabia after all remarks had been agreed upon.

## 3. Results

The guidelines for complications came up from systematic reviews and meta-analysis of American guidelines, and the third draft of guidelines was subjected to three rounds of the modified Delphi method.

During the first round, a total of 37 statements were circulated among 14 panel members through email, asking about their opinions regarding each statement. The database was used by the research assistants to anonymously record and calculate scores for each statement. The comments that were approved by 80% of the experts were used and included in the final documents, while the remainder were altered and allocated for round 2, and experts were also encouraged to provide better statements where necessary. A total of 15 statements were approved by over 80% of the panellists during round 1, and the remaining 22 were emailed back to them for review after modification based on the comments and feedback during this round for consensus building during the next round.

During the second round, the panellists were encouraged to debate as to why they agreed or disagreed with the remaining 22 statements. Final replies were examined as indicated in round 1, and comments that did not exceed the 80 percent threshold were distributed once more in round 3 for discussion among the experts. During this round, 13 statements received acceptance from more than 80% of the panellists, while the remaining 9 were circulated again for round 3.

**Figure 1:**
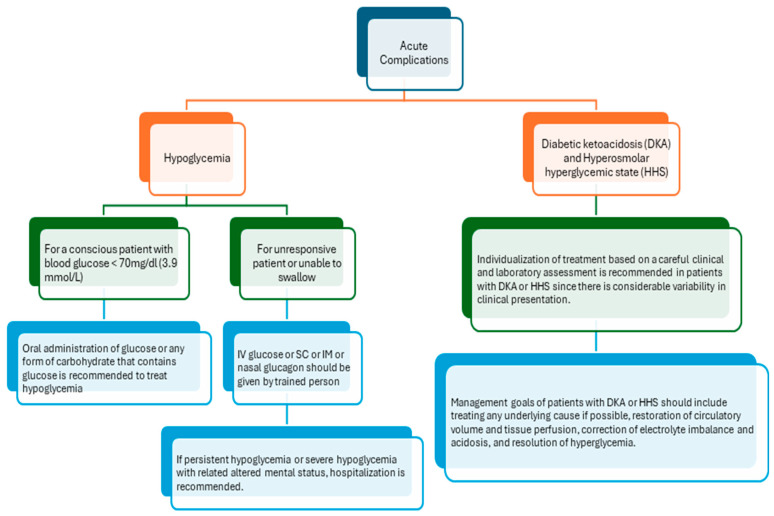
Algorithm for the management of diabetes mellitus (DM) acute complications.

**Figure 2:**
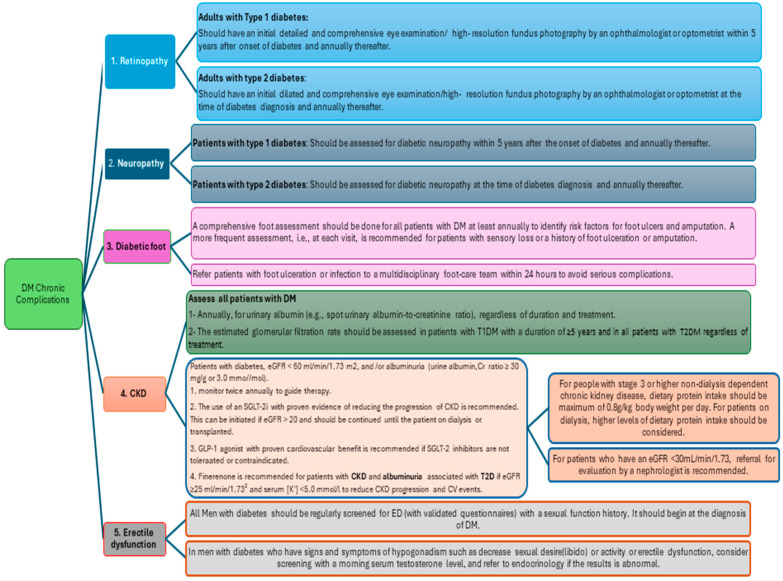
Management algorithm for diabetes mellitus (DM) chronic complications.

During the last round, the remaining nine statements were subject to debate among the panellists in a face-to-face meeting, and after a debate followed by some modifications, a consensus was achieved for the remaining nine statements. The details of the recommendations for DM acute and chronic complications are depicted in Figure 1 and Figure 2.

### 3.1. Acute Complications

#### 3.1.1. Hypoglycemia

The final guidelines for the management of acute complications of diabetes are explained in Figure 1 and Table 1. For hypoglycemia, the guidelines included nine recommendations (Table 1) around the management of hypoglycemia complications. It was recommended that at every medical appointment, the risk of hypoglycemia should be evaluated for individuals with diabetes. If the patient is conscious and has low blood sugar levels (less than 70 mg/dL), they should be given 15 g oral glucose or a carbohydrate source. It is important to have glucagon available for patients at increased risk of level 2 or 3 hypoglycemia, and if the patient is unresponsive or unable to swallow, intravenous glucose or glucagon should be given by a trained person. Furthermore, hospitalization is necessary for patients experiencing persistent or severe hypoglycemia with altered mental function. After a hypoglycemia episode, education and therapy adjustments are recommended, and strict avoidance of hypoglycemia is advised. Finally, it was recommended that regular assessments and medical identification are important for patients with cognitive impairment.

#### 3.1.2. Diabetic Ketoacidosis (DKA) and Hyperosmolar Hyperglycemic State (HHS)

For Diabetic Ketoacidosis (DKA) and Hyperosmolar Hyperglycemic State (HHS) conditions, two recommendations were included in the guideline. It was recommended that individualized treatment be developed based on a thorough clinical and laboratory evaluation. The main objectives of managing these conditions should include addressing the underlying causes, improving blood flow and oxygenation to the tissues, fixing imbalanced electrolytes and acid levels, and controlling high blood sugar. These goals must be achieved to ensure effective and successful treatment.

### 3.2. Chronic Complications

The final guidelines for the management of acute complications of diabetes are explained in Figure 2 and Table 1.

#### 3.2.1. Diabetic Retinopathy

For the management of diabetic retinopathy, a chronic complication of diabetes, a total of five recommendations were included in the guideline.

It was recommended that to prevent or slow the progression of diabetic retinopathy, it is important to properly manage glycemic control, blood pressure, and serum lipid levels. For patients with diabetic macular edema, proliferative diabetic retinopathy, or non-proliferative diabetic retinopathy of moderate or worse level, it is recommended to promptly seek the assistance of an experienced ophthalmologist. Furthermore, individuals with type 1 diabetes mellitus (T1DM) should have their first comprehensive eye examination within 5 years of their diabetes diagnosis and annually thereafter. In addition, patients with T2DM should have an initial comprehensive eye examination at the time of their diabetes diagnosis and annually thereafter. Finally, aspirin therapy for cardio protection should not be avoided even if the patient has retinopathy, as aspirin does not increase the risk of retinal hemorrhage.

#### 3.2.2. Diabetic Peripheral Neuropathy

For the management of diabetic peripheral neuropathy, the guidelines included five recommendations. It was recommended that patients with T2DM should undergo assessment starting at diagnosis, while patients with T1DM should undergo assessment 5 years after diagnosis and at least annually thereafter. Assessment should include a careful history and examination of temperature or pinprick sensation and vibration sensation using a 128 Hz tuning fork. Annual 10 g monofilament testing is also recommended to identify feet at risk for ulceration and amputation. Proper glycemic control is crucial in preventing or slowing down the progression of neuropathy. For neuropathic pain, pregabalin, duloxetine, or gabapentin are the recommended initial treatments. However, a tailored and stepwise therapeutic approach, with attention to symptom improvement, medication adherence, and side effects, is recommended for better pain reduction and quality of life, as available treatments are only partially effective.

#### 3.2.3. Diabetic Foot

For the management of diabetic foot, the guidelines included five recommendations. For the proper management of diabetic foot, it was recommended that patients with diabetes should undergo annual assessments to identify any risk factors for foot ulcers and amputation. If a patient has a history of foot ulceration, amputation, or sensory loss, they should be assessed more frequently. The assessment should include a comprehensive review of the patient’s foot-related history, symptoms, and physical examination. This examination should look for any visual signs of neuropathy or ischemia, foot deformities, and the patient’s footwear and pulse status. Based on the results of the assessment, patients should receive individualized foot-care education and a foot-care plan. If a patient smokes or has a history of lower-extremity complications, peripheral arterial disease, or structural abnormalities, a referral to a foot-care specialist is recommended. Finally, it was also recommended that if a patient has a foot ulcer or infection, they should be referred to a multidisciplinary foot-care team within 24 h to avoid serious complications.

#### 3.2.4. Chronic Kidney Disease

A total of nine recommendations were provided in the guideline for the management of chronic kidney diseases. It was recommended that diabetic patients undergo annual assessments of urinary albumin levels and an estimated glomerular filtration rate. If urinary albumin/creatinine ratio is ≥30 mg/g (≥3.0 mmol/mol) and/or an estimated glomerular filtration rate is between <60 mL/min/1.73 m^2^, the patient should be monitored twice a year. For patients with T2DM and urinary ACR ≥ 30 mg/g (≥3.0 mmol/mol), a sodium–glucose cotransporter 2 inhibitor with proven evidence of reducing the progression of CKD is recommended. This can be initiated if eGFR > 20 and should be continued until the patient on dialysis or transplanted. A GLP-1 agonist with proven cardiovascular benefit is recommended if SGLT-2 inhibitors are not tolerated or contraindicated. A nonsteroidal mineralocorticoid receptor antagonist is recommended for patients with CKD and persistent albuminuria despite the maximally tolerated dose of ACE inhibitors/ARBs and SGLT-2 inhibitors if eGFR ≥25 mL/min/1.73^2^ and serum [K+] <5.0 mmol/L to reduce the risk of chronic kidney disease progression and cardiovascular events. To further reduce the risk or slow progression, blood pressure and glucose control should be optimized. Patients with stage 3 or higher non-dialysis-dependent chronic kidney disease should limit protein intake to 0.8 g/kg body weight per day, while patients on dialysis may require a higher intake. For patients with diabetes and hypertension, either an ACE inhibitor or angiotensin receptor blocker is recommended. If the estimated glomerular filtration rate is <30 mL/min/1.73 m^2^, a referral to a nephrologist is necessary.

#### 3.2.5. Erectile Dysfunction

Finally, for the management of erectile dysfunction, a chronic disease of DM, two recommendations were provided in the guideline. It was recommended that all men with diabetes should be regularly screened for erectile dysfunction starting at diagnosis and using a sexual function history questionnaire (e.g., International Index of Erectile Function or Sexual Health Inventory for Men). If the man has symptoms or signs of hypogonadism, such as decreased libido or sexual activity or ED, a morning serum testosterone level should be considered for screening, and a referral to endocrinology made if the result is abnormal.

## 4. Discussion

As a result of these discussions, a formulation of guidelines was implemented, which would aid in the evidence-based management of prevention and treatment of diabetes complications. The findings in accordance with this study suggest that individuals who are taking drugs that lower glucose need to regularly monitor their glucose [31]. On the other hand, individuals with diabetes who take medications that do not affect blood glucose levels are not usually required to self monitor. For all diabetics, it is important to schedule regular consultations with diabetologists to ensure that their blood glucose is effectively managed and their condition is regularly monitored. Additionally, nighttime blood glucose monitoring is also recommended for diabetics. There is a need for risk stratification of diabetics in terms of identifying the individuals who have already suffered from hypoglycemic episodes, as these individuals are more likely to suffer from such episodes in the future. It is important to educate these individuals on the use of glucagon in an emergency. Additionally, these individuals are also required to be educated about different tools that are useful for self-monitoring their own blood glucose. This management of blood glucose becomes challenging in individuals who are cognitively impaired, and different strategies need to be adapted to deal with issues specific to these individuals. These strategies may vary from case to case depending on the type of cognitive disability the person has and the severity of the condition.

This study’s discussion resulted in recommendations that were aligned with the recommendations of the American Diabetes Association [32] and the International Diabetes Federation, which included a healthy and balanced lifestyle with an emphasis on introducing physical activity as part of a daily routine. Additionally, it was recommended that diabetic patients stick to the medications and hypoglycemic agents suggested to them by their diabetologists to ensure the effective management of blood glucose levels. While hyperglycemia poses significant risks in diabetics, the discussion also emphasized the importance of recognizing and treating hypoglycemia, which can have a negative impact on virtually every organ system. Additionally, it can substantially affect the quality of life of the individual, resulting in compromised sleep routines and problems in daily activities like driving and walking. Therefore, to avoid hypoglycemia episodes, it is important to personalize the treatment to an individual, ensuring that individual characteristics of the patient are kept in mind while prescribing different treatment regimens [33].

Data from our investigations found a concerning prevalence of painful diabetic peripheral neuropathy (PDPN) in 29.8% of type 2 diabetic patients in Saudi Arabia. This emphasizes the critical need for effective management strategies, which potentially include pain medications, to alleviate suffering and prevent further complications [34]. This is quite concerning because a staggering one-third of diabetic patients are either unaware of their diagnosis or disregard treatment, resulting in neuropathy. It is important to ensure that patients suffering from diabetes are made aware of the potential consequences of the suboptimal management of their diabetes in clear and explicit ways and assert to them the need to effectively manage their glucose [35]. Regular diabetologist consultations can serve as a foundation for proactive risk education on potential risks to avoid complications like diabetic neuropathy. According to a recent study, Jeddah has 325 amputations per year, while Riyadh has 741 due to diabetic complications [36]. This is more evidence in favor of the fact that diabetes management needs to be improved at the level of the population to ensure the effective prevention of complications. Additionally, erectile dysfunction is an important complication of diabetes. Effectively managing erectile dysfunction is critical to ensuring sexual health and avoiding potential problems caused by sexual frustration.

The implementation of guidelines for managing type 2 diabetes poses several issues. These include diversity in healthcare resources, financial scarcity, and variability in infrastructure. Additionally, offering healthcare provider training and overcoming resistance to change, coupled with patient adherence, is influenced by factors including health literacy and cultural beliefs. For a smooth adoption process, it is essential to incorporate electronic health records and efficient communication techniques. In order to ensure continuous improvement in the delivery of diabetes treatment, successful adoption will also require a collaborative strategy, including healthcare providers, legislators, educators, and patients, as well as continuing reviews to identify and remove impediments.

Addressing potential gaps in the current management guidelines, such as limited interdisciplinary collaboration, unclear patient participation, reliance on specific resources, lack of generalizability, and recommendations based on evidence of varying quality, could benefit it. These drawbacks highlight the need for ongoing assessment, adaptation, and inclusion to guarantee the effectiveness of the guideline across a range of healthcare environments and patient demographics.

## 5. Conclusions

The essence of these guidelines is early detection, treatment, and using contextual evidence to manage diabetes in Saudi Arabia. For the effective prevention of diabetes-related complications, it is important that the relevant stakeholders create mechanisms to collaborate effectively. Though these guidelines provide an overarching approach to managing diabetes and its complications, adapting these in other Middle Eastern countries will require the generation of contextual evidence considering the differences in the local cultures as well as the differences in the health systems of other countries.

## Figures and Tables

**Table 1:** Recommendation statements for diabetes mellitus (DM) complications.

**Statements**	**Reference**	**LOE**
**I. ACUTE COMPLICATIONS**		
**Hypoglycemia**		
1. At every visit, the incident, symptoms, and risks for hypoglycemia should be discussed and investigated with individuals with diabetes.	ADA (2021) [22]	C
2. For a conscious patient with blood glucose <70 mg/dL (3.9 mmol/L), oral administration of glucose or any form of carbohydrate that contains glucose is recommended to treat hypoglycemia.	ADA (2021) [22]	B
3. For any patient with an increased risk of level 2 or 3 hypoglycemia, glucagon should be available to be used whenever needed.	ADA (2021) [22]	E
4. For an unresponsive patient or unable to swallow, IV glucose or SC, IM, or nasal glucagon should be given by a trained person.	AACE/ACE (2023) [23]	Grade A; BEL 1
5. For patients with persistent hypoglycemia or severe hypoglycemia with altered mental status, hospitalization is recommended.	AACE/ACE (2023) [23]	Grade A; BEL 1
6. Education on hypoglycemia avoidance, adjustment of therapy, and reevaluation are recommended if ≥1 episode of level 3 hypoglycemia or hypoglycemia unawareness.	ADA (2021) [22]	E
7. Strict avoidance of hypoglycemia by raising the glycemic targets for at least several weeks is recommended in patients on insulin with 1 episode of level 3 hypoglycemia, level 2 hypoglycemia of an unexplained pattern, or hypoglycemia unawareness to reduce the risk of future episodes.	ADA(2021) [22]	A
8. For patients with cognitive impairment, regular assessment is advised with better attentiveness for hypoglycemia.	ADA (2021) [22]	B
9. Patients should be encouraged to wear medical identification that tells others they have DM (bracelet or necklace). Then, people may be better able to properly help in any emergency situation.	Expert opinion	E
**Diabetic ketoacidosis (DKA) and hyperosmolar hyperglycemic state (HHS)**		
10. Individualization of treatment based on a careful clinical and laboratory assessment is recommended in patients with DKA or HHS since there is considerable variability in clinical presentation.	Kitabchi, Vellanki, (2017) [24] Harrison, Hsia (2017) [25]	C
11. Management goals of patients with DKA or HHS should include treating any underlying cause, if possible, restoration of circulatory volume and tissue perfusion, correction of electrolyte imbalance and acidosis, and resolution of hyperglycemia.	Fayfman (2019) [26]	E
**II. CHRONIC COMPLICATIONS**		
**Diabetic retinopathy**		
12. Proper optimization of glycemic control, blood pressure, and serum lipid is recommended to minimize the risk of diabetic retinopathy or slow down its progression.	ADA (2021) [22]	A
13. Patients with diabetic macular edema (of any level), proliferative diabetic retinopathy (of any level), or non-proliferative diabetic retinopathy (moderate or worse) should be promptly referred to an experienced ophthalmologist.	ADA (2021) [22]	A
14. Adults with T1DM should have an initial dilated and comprehensive eye examination/high-resolution fundus photography by an ophthalmologist or optometrist within 5 years after the onset of diabetes and annually thereafter.	Solomon (2019) [27]	B
15. Patients with T2DM should have an initial dilated and comprehensive eye examination/high-resolution fundus photography by an ophthalmologist or optometrist at the time of the diabetes diagnosis and annually thereafter.	Solomon (2019) [27]	B
16. The presence of retinopathy is not a contraindication to aspirin therapy for cardioprotection, as aspirin does not increase the risk of retinal hemorrhage.	Solomon (2019) [27]	A
**Diabetic neuropathy**		
17. All patients should be assessed for diabetic peripheral neuropathy starting at diagnosis of T2DM and 5 years after the diagnosis of T1DM and at least annually thereafter.	ADA (2021) [22]	B
18. Assessment for distal symmetric polyneuropathy should include a careful history and assessment of either temperature or pinprick sensation (small fiber function) and vibration sensation using a 128 Hz tuning fork (for large-fiber function). All patients should have annual 10 g monofilament testing to identify feet at risk for ulceration and amputation.	ADA (2021) [22]	B
19. Proper optimization of glycemic control should be maintained to prevent, delay, or slow the progression of neuropathy in patients with DM.	ADA (2021) [22]	A&B
20. Pregabalin, duloxetine, or gabapentin are the recommended initial treatments for neuropathic pain in diabetes.	ADA (2021) [22]	A
21. Since the available treatment options are considered partially effective, a tailored and stepwise therapeutic strategy with an attentive observation of symptom improvement, medication adherence, and side effects is recommended to achieve pain reduction and improve quality of life.	Bril, Griebeler, Ziegler (2022) [28]	E
**Diabetic foot**		
22. All patients with DM should be assessed at least annually to identify risk factors for foot ulcers and amputation. A more frequent assessment, i.e., at each visit, is recommended for patients with sensory loss or a history of foot ulceration or amputation.	ADA (2021) [22]	B
23. A comprehensive foot assessment should include the history of previous foot ulceration or amputation, symptoms of peripheral arterial disease, physical or visual difficulty in self-foot-care, foot deformity (hammer or clawed toes, bone prominences), visual evidence of neuropathy (dry skin, dilated veins) or incipient ischemia; callus; nail deformity or damage; footwear, palpation of foot pulses (dorsalis pedis and posterior tibial).	IDF (2022) [29]	E
24. An individualized foot-care education and a foot-care plan should be given to the patient based on the findings of the foot assessment and according to the individual needs and risks.	IDF (2022) [29]	E
25. For patients who smoke or who have a history of prior lower-extremity complications, peripheral arterial disease, loss of protective sensation, or structural abnormalities, referral to a foot care specialist is recommended.	ADA (2021) [22]	C
26. Patients with foot ulceration or infection should be referred to a multidisciplinary foot-care team within 24 h to avoid serious complications.	IDF (2022) [29]	E
**Chronic kidney disease**		
27. All patients with DM should be assessed at least annually for urinary albumin (e.g., spot urinary albumin-to-creatinine ratio).	ADA (2021) [22]	B
28. The estimated glomerular filtration rate should be assessed in patients with T1DM with a duration of ≥5 years and in all patients with T2DM, regardless of treatment.	ADA (2021) [22]	B
29. Patients with diabetes and urinary albumin/creatinine ratio ≥ 30 mg/g creatinine and/or an estimated glomerular filtration rate of <60 mL/min/1.73 m^2^ should be monitored twice annually to guide therapy.	ADA (2021) [22]	B
30. For patients with T2DM and an eGFR ≥ 20 mL/min/1.73 m^2^ and urinary albumin/Cr ratio ≥ 30 mg/g (≥3.0 mmol/mol), the use of a sodium–glucose cotransporter 2 inhibitor is recommended to reduce the progression of chronic kidney disease progression and cardiovascular events. If unable to use a sodium–glucose cotransporter 2 inhibitor, a GLP-1 agonist with proven cardiovascular benefit is recommended. 31. Nonsteroidal mineralocorticoid receptor antagonist (finerenone) is recommended for patients with CKD and persistent albuminuria associated with T2D despite treatment of maximally tolerated ACE inhibitors or ARBs and SGLT inhibitors if eGFR ≥25 mL/min/1.73^2^ and serum [K+] <5.0 mmol/L to reduce CKD progression and CV events.	ADA (2024) [30]	A
32. Optimization of blood pressure and glucose control is recommended to reduce the risk or slow the progression of chronic kidney disease.	ADA (2021) [22]	A
33. For people with stage 3 or higher non-dialysis-dependent chronic kidney disease, dietary protein intake should be a maximum of 0.8 g/kg body weight per day. For patients on dialysis, higher levels of dietary protein intake should be considered.	ADA (2021) [22]	A&B
34. For patients with diabetes and hypertension who are non-pregnant, either an ACE inhibitor or an angiotensin receptor blocker is recommended.	ADA (2021) [22]	A&B
35. For patients who have an eGFR < 30 mL/min/1.73, referral for evaluation by a nephrologist is recommended.	ADA (2021) [22]	A
**Erectile dysfunction**		
36. All men with diabetes should be regularly screened for ED with a sexual function history. It should begin at the diagnosis of DM.	Expert opinion	E
37. Validated questionnaires (e.g., International Index of Erectile Function or Sexual Health Inventory for Men) can be used in determining the presence of ED and assessing response to treatment.	Expert opinion	E
38. In men with diabetes who have symptoms or signs of hypogonadism, such as decreased sexual desire (libido) or activity or erectile dysfunction, consider screening with a morning serum testosterone level and refer to endocrinology if the result is abnormal.	ADA (2021) [22]	B

## Data Availability

No new data were created or analyzed in this study. Data sharing is not applicable to this article.
